# Health-related quality of life of a conflict-affected population in Colombia

**DOI:** 10.1007/s11136-021-02805-5

**Published:** 2021-04-10

**Authors:** Fan Yang, Sebastian Leon-Giraldo, Rodrigo Moreno-Serra

**Affiliations:** 1 Centre for Health Economics, University of York, York, UK; 2 Alberto Lleras Camargo School of Government and Interdisciplinary Centre of Development Studies, University of Los Andes, Bogotá, Colombia

**Keywords:** Health-related quality of life, EQ-5D, Validity, Conflict, Peace accord, Colombia

## Abstract

**Purpose:**

We assessed the validity of the EQ-5D instrument; explored correlations between area of residence’s conflict intensity and individual health-related quality of life (HRQoL); and identified factors associated with HRQoL in a conflict-affected population in Colombia.

**Methods:**

We conducted a household survey among residents of the Meta province, collecting longitudinal information about HRQoL (EQ-5D-3L instrument), health, demographic and socio-economic indicators, for years 2014 (pre-2016 peace accord), 2018 (post-peace accord) and 2019 (follow-up). After examining EQ-5D’s validity, we analysed panel data using multivariate random effects models to explore associations between area conflict levels (and other factors) and HRQoL. We scrutinised these results further through multivariate linear regressions using cross-sectional data, and provided preliminary estimates of quality-adjusted life years (QALYs) gained since the Colombian peace accord.

**Results:**

In total, 1309 individuals provided information for years 2014 and 2018; 1106 individuals were followed-up in 2019. Mean EQ-5D scores in 2014, 2018 and 2019 were 0.898, 0.846 and 0.902, respectively. The tests confirmed the validity of EQ-5D. Our estimations indicated a dose–response relationship between conflict levels and HRQoL: people in lightly and heavily affected areas had 0.019 and 0.037 lower EQ-5D scores (respectively) than people in non-affected areas. Other relevant factors included age, marital status, education, assets and health status. We estimated QALY gain of 0.0343 per individual and 20,752 for all Meta adults since the peace accord.

**Conclusion:**

We found EQ-5D to be a valid instrument for HRQoL measurement in a conflict-affected population. Area conflict intensity was negatively associated with individual HRQoL.

**Supplementary Information:**

The online version contains supplementary material available at 10.1007/s11136-021-02805-5.

## Introduction

Armed conflicts have profound adverse effects on people’s health [1]. These effects usually last for long periods in the aftermath of armed conflicts, also due to the destruction of health-supporting infrastructure [2]. This is particularly the case in countries beset by protracted civil confrontations, which have become the norm in recent decades [3]. Policymakers need a good understanding of the consequences of conflicts for people’s health and wellbeing, in order to identify the most vulnerable groups and guide actions to meet their needs. Yet related research has focused mostly on specific aspects such as increased morbidity patterns [4, 5] and adverse effects on mental health [6, 7]. There has been scarce assessment of the health consequences of conflict using broader measures of health-related quality of life (HRQoL) which can, combined with standard measures (e.g., mortality and clinical indicators), offer a more comprehensive picture of such impact.

HRQoL is a multi-dimensional concept that captures information related to physical, mental, emotional, and social well-being [8]. It has been used by clinicians, researchers and public health officials to measure the effects of diseases and treatments [9, 10]. In the literature about conflict-affected populations, HRQoL has been assessed most frequently through the Short-Form Health Survey 36-item (SF-36) [11–13] and the World Health Organisation Quality of Life-BREF (WHOQOL-BREF) [14, 15]. To the best of our knowledge (and perhaps surprisingly), the instrument most commonly used in the general HRQoL literature, EQ-5D [16], has not been used among conflict-affected people, and evidence is lacking about its validity to measure HRQoL in these populations. As EQ-5D provides utility scores for cost-effectiveness analysis to inform healthcare resource allocation [17], knowledge about its validity could also contribute to priority setting regarding healthcare interventions for conflict-affected people.

For over five decades, Colombia endured a civil conflict that killed more than 220,000 people and displaced more than 7 million (13% of the population) [18], before a peace accord between FARC (the main rebel group) and the Colombian government was signed in December 2016. Although we could expect the peace accord to have had a positive impact on population health, empirical evidence about the HRQoL of conflict-affected people in Colombia, either before or after the peace accord, is completely absent [19]. Filling the knowledge gaps about the consequences of the long-term conflict for people’s HRQoL, and how HRQoL changed after the peace accord, can provide valuable insights for the development of effective post-conflict health policies in Colombia and other countries affected by protracted violence.

Our study has three aims:

1. To assess the validity of EQ-5D in a conflict-affected population;
2. To explore the correlations between conflict intensity levels in the area of residence of individuals and their HRQoL, using longitudinal individual-level data referring to periods before and after the peace accord in Colombia;
3. To identify factors associated with HRQoL among conflict-affected individuals in Colombia.

## Methods

### Data

Data used in this study were from two rounds of a household survey (Conflict, Peace and Health survey—CONPAS), which was developed by the authors within a larger research project. CONPAS was applied to a representative sample of households from all 29 municipalities of the Meta province, Colombia (one adult respondent per household). The Meta province was affected by the armed conflict from an early stage and these municipalities were exposed to different degrees of conflict violence prior to the 2016 peace accord. This enables us to compare residents of areas with similar socio-economic and demographic profiles, yet different exposure to the conflict, thus mitigating the influence of confounders in our analyses.

Respondents completed the questionnaire administered by an interviewer in a home visit. CONPAS modules included information about demographic characteristics (age, sex, ethnicity, marital status, education level, occupation) and socio-economic status (urban/rural location, assets, health insurance). We also collected information about several health indicators and risk factors, including whether the respondent was sick or hospitalised in the last 12 months; hazardous alcohol consumption assessed using the Alcohol Use Disorders Identification Test (AUDIT) [20]; smoking dependence assessed using the Fagerstrom test [21]; general self-reported health using a 5-point scale (excellent/very good/good/fair/poor); mental health using the WHO Self-Reporting Questionnaire (SRQ-20, with eight or more positive responses indicating risk of presenting a mental health disorder) [22]; disability using the WHO Disability Assessment Schedule 2.0 (WHODAS 2.0, with a score of 17 or higher indicating disability) [23]; and HRQoL using the 3-level EQ-5D (EQ-5D-3L) [16].

The first survey round was conducted in 2018 (post-peace accord). Respondents provided answers regarding their current condition (year 2018) and their condition during year 2014 (pre-peace accord). For the latter, respondents were instructed to recall their life in 2014, with the help of information about major events that occurred in that year, e.g., the national presidential elections and the FIFA World Cup. The questions referring to year 2014 were confirmed with a second respondent in the household whenever possible to help with recall. One year later, we conducted a follow-up survey asking the same respondents about their condition in 2019 (follow-up). In the 2019 survey, we also implemented recall tests based on: (a) a selection of 17 CONPAS questions referring to year 2014, that were asked again in 2019 from all respondents, to be compared with the responses provided in the 2018 CONPAS round; and (b) implementation of a Flashbulb Test [24], where we asked questions about a common and known past event in order to assess deviations from the right responses. We found for both tests that recall error patterns are uncorrelated with the level of conflict in the area of residence, offering reassurance that any recall error in the 2014 survey responses is likely to be randomly distributed in our sample—and thus unlikely to confound the estimated associations in our analyses.

The level of conflict violence in the respondent’s municipality of residence was defined using the Colombian Conflict Analysis Resource Centre (CERAC) classification [25]. This classification is based on the presence of armed groups and frequency of conflict violence episodes between 2000 and 2012, including seven groups. In this study, we consolidated the groups into three and assigned each municipality to one of three groups: “heavily affected”, “lightly affected” or “not affected” by conflict violence.

### Instrument

The EQ-5D instrument has 5 items (mobility, self-care, usual activities, pain/discomfort, and anxiety/depression), with multiple descriptive levels for each item to enable respondents to choose the appropriate level based on their health condition on the day of the survey [16]. For our survey, we used the EQ-5D-3L with 3 levels for each item (no, some and extreme problems) [16]. Responses to the five items define a health state and a summary index score can be calculated using the ‘value set’ which provides values for each health state according to the preferences of the general population of a country/region [16]. The index score is anchored by 0 (death) and 1 (full health), with higher scores corresponding to better HRQoL.

### Analysis

As there is no EQ-5D-3L value set available for Colombia, we used the value set for Argentina [26] to calculate the EQ-5D index scores. The characteristics of people included in our study were described using means and standard deviations (SDs) for continuous variables, and frequencies and percentages for categorical variables.

First, we assessed the validity of the EQ-5D instrument for our study population by testing correlations between EQ-5D scores and other health measures (general health, mental health and disability), as well as examining whether EQ-5D scores could distinguish between groups that are theoretically expected to differ in their HRQoL (e.g., young vs. old individuals). We hypothesised that the correlations would be moderate to strong with coefficients ≥ 0.4, and that people with better underlying health status (e.g., younger or healthier) would have statistically significant higher EQ-5D scores than their counterparts.

Second, we analysed our panel data (2014, 2018 and 2019) using multivariate random effects models to explore the association between conflict level in the area of residence of an individual and their HRQoL, accounting for the demographic, socioeconomic and health-related characteristics. The random effects approach with panel data is more appropriate for our study aims than cross-sectional analysis because: (i) it provides information about the dynamics of people’s HRQoL during and after conflict; (ii) it allows exploration of the associations over time between HRQoL and time-invariant characteristics (e.g., pre-accord conflict level in the person’s municipality of residence; sex and ethnicity); and (iii) it mitigates the influence of confounders of the associations of interest, by accounting for changes over time on several individual characteristics and for correlation over time in these characteristics. We also explored potential heterogeneity in the relationship between conflict levels and HRQoL for different population subgroups.

Third, we extended the analysis by applying multivariate linear regression models to the cross-sectional data (i.e., performing separate analyses for years 2014, 2018 and 2019) to investigate the association between conflict intensity and HRQoL within a given year, to derive further insights for the interpretation of the dynamics of HRQoL suggested by the panel data analyses.

Fourth, we re-ran our panel data analysis using EQ-5D scores calculated using value sets available for other countries in South America, namely Brazil [27] and Chile [28], to determine whether the conclusions were consistent with those in the main analysis.

Finally, we used the EQ-5D scores in 2014, 2018 and 2019 to obtain a preliminary estimate of the gain in quality-adjusted life years (QALYs) for the study population since the Colombian peace accord, based on the area under the curve method and linear interpolation between time points [29]. We assumed that in the counterfactual scenario of there being no peace accord, people’s HRQoL would have continued to follow the same downward trend from 2018 to 2019, as it did from 2014 to 2018. We estimated QALY gain for each individual in the sample, and then extrapolated such gain to the estimated 605,000 adults in Meta to obtain a back-of-the-envelope measure of the total QALY gain for the province’s adult population.

## Results

### Sample

The full description of demographic, socioeconomic and health-related characteristics for each year is shown in Table 1. A total of 1309 individuals provided complete information for years 2018 and 2014. In year 2018, the mean (SD) age was 46.5 (16.5) years, with 31.1% aged 55 year above, 45.8% male, 42.9% White, 62.4% married or co-habiting, 46.9% with primary or lower education, 35.9% employed and 26.6% living in the rural areas. In the previous 12 months, a minority were hospitalised (12.4%) but more were sick (59.9%); the majority consumed alcohol at a non-hazardous level (88.2%) and smoking dependence was low for most respondents (98.0%). For health indicators, nearly half of people reported good, very good or excellent health (46.5%), 67.6% did not present risk of mental health disorder, and 79.9% had no disability. In terms of conflict level, 54.2% of people were living in the lightly affected municipalities and 23.4% in the heavily affected municipalities. In the follow-up survey, 1106 individuals provided complete information. Mean characteristics were broadly similar to those in the first survey round despite this loss to follow-up, although there was a slightly lower proportion of people living in the lightly affected municipalities in the follow-up survey (51.5% vs. 54.2%) (Table S1).

**Table 1:** Characteristics of respondents in years 2014, 2018 and 2019

	2014 (*n* = 1309)		2018 (*n* = 1309)		2019 (*n* = 1106)	
Age, mean (SD)	42.5	(16.5)	46.5	(16.5)	48.4	(16.2)
Age group, *n* (%)						
≤ 30	370	(28.3)	289	(22.1)	193	(17.5)
31–55	630	(48.1)	613	(46.8)	532	(48.1)
> 55	309	(23.6)	407	(31.1)	381	(34.4)
Male, *n* (%)	600	(45.8)	600	(45.8)	509	(46.0)
Ethnicity, *n* (%)						
White	562	(42.9)	562	(42.9)	473	(42.8)
Black/Mestizo	217	(16.6)	217	(16.6)	192	(17.4)
Others	530	(40.5)	530	(40.5)	441	(39.9)
Marital status, *n* (%)						
Married/co-habiting	848	(64.8)	817	(62.4)	735	(66.5)
Single	169	(12.9)	99	(7.6)	83	(7.5)
Separated/divorced/widowed	292	(22.3)	393	(30.0)	288	(26.0)
Education, *n* (%)						
Primary/lower	643	(49.1)	614	(46.9)	670	(60.6)
Secondary	454	(34.7)	439	(33.5)	259	(23.4)
Technical or higher	212	(16.2)	256	(19.6)	177	(16.0)
Occupation, *n* (%)						
Employed	586	(44.8)	470	(35.9)	417	(37.7)
Self-employed	291	(22.2)	314	(24.0)	258	(23.3)
Unemployed/others	432	(33.0)	525	(40.1)	431	(39.0)
Urban, *n* (%)						
Rural	348	(26.6)	348	(26.6)	299	(22.8)
Urban/town	961	(73.4)	961	(73.4)	1010	(77.2)
Assets tertile, *n* (%)						
Poor	492	(37.6)	437	(33.4)	369	(33.4)
Middle	382	(29.2)	438	(33.5)	369	(33.4)
Rich	435	(33.2)	434	(33.2)	368	(33.3)
Health insurance, *n* (%)						
EPS (contributory)	410	(31.3)	350	(26.7)	323	(29.2)
EPS (subsidised)	771	(58.9)	829	(63.3)	719	(65.0)
No insurance/others	128	(9.8)	130	(9.9)	64	(5.8)
Hospitalisation in the previous 12 m, *n* (%)	162	(12.4)	162	(12.4)	135	(12.2)
Sickness in the previous 12 m, *n* (%)	581	(44.4)	784	(59.9)	613	(55.4)
Alcohol consumption, *n* (%)						
Normal use	1107	(84.6)	1155	(88.2)	1016	(91.9)
Hazardous use	202	(15.4)	154	(11.8)	90	(8.1)
Smoking, *n* (%)						
Low dependence	1256	(96.0)	1283	(98.0)	1083	(97.9)
Moderate to high dependence	53	(4.0)	26	(2.0)	23	(2.1)
General health, *n* (%)						
Poor	69	(5.3)	123	(9.4)	73	(6.6)
Fair	350	(26.7)	577	(44.1)	516	(46.7)
Good/very good/excellent	890	(68.0)	609	(46.5)	517	(46.8)
Mental health disorder, *n* (%)						
< 8, no	1111	(84.9)	885	(67.6)	791	(71.5)
≥ 8, yes	198	(15.1)	424	(32.4)	315	(28.5)
Disability, *n* (%)						
< 17, no	1146	(87.6)	1046	(79.9)	933	(84.4)
≥ 17, yes	163	(12.4)	263	(20.1)	173	(15.6)
Conflict level, *n* (%)						
Not affected	–		294	(22.5)	–	
Lightly affected	–		709	(54.2)	–	
Heavily affected	–		306	(23.4)	–	

**Fig. 1:**
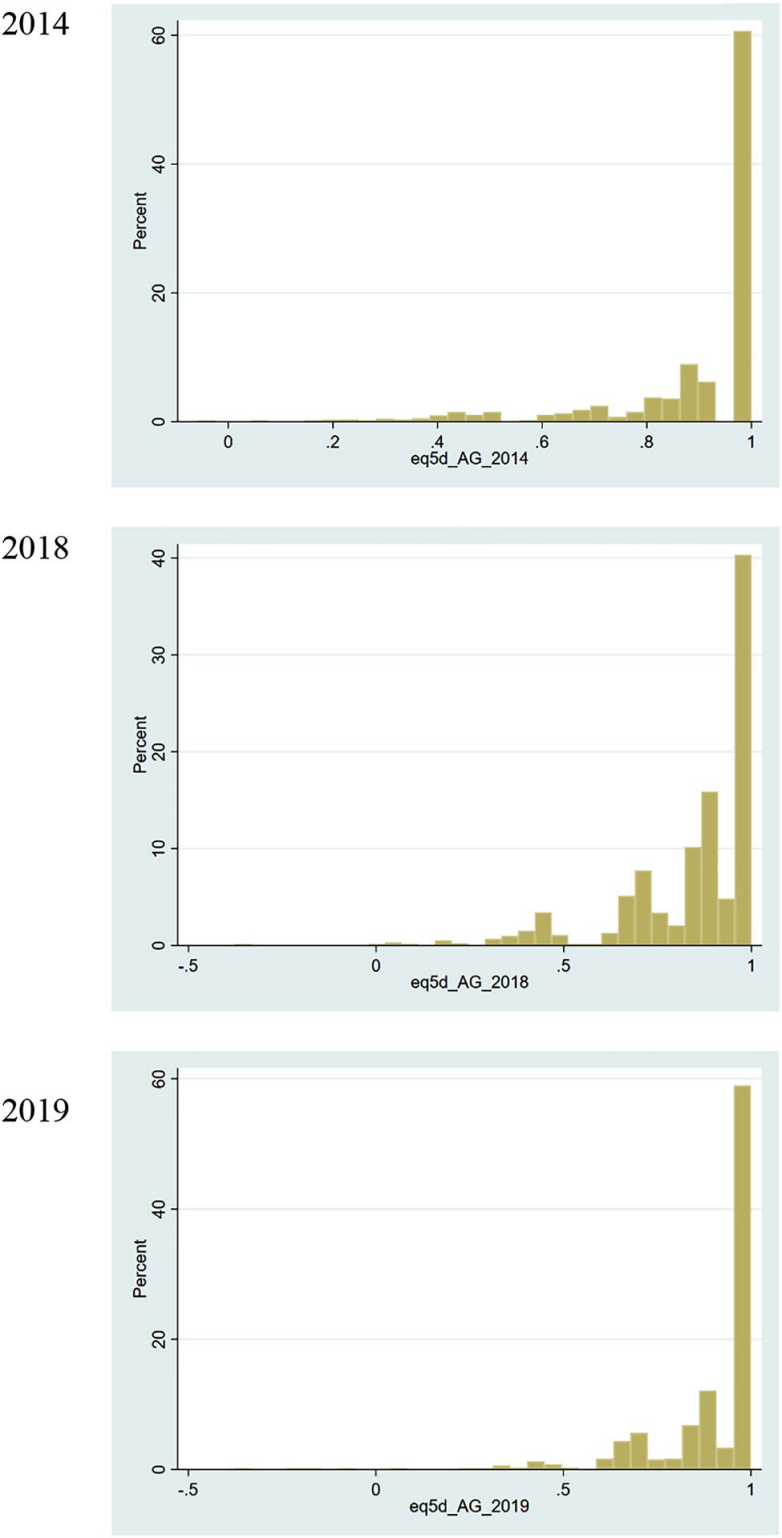
Distribution of EQ-5D scores by year

Mean (SD) EQ-5D scores in 2014, 2018 and 2019 were 0.898 (0.178), 0.846 (0.191) and 0.902 (0.163), respectively (Table 2). The distributions were highly skewed (Fig. 1) with 60.7%, 40.3% and 59.0% of respondents reporting full health in 2014, 2018 and 2019 (Table 2), indicating the presence of ceiling effect. People living in the heavily conflict-affected municipalities had statistically significant lower EQ-5D scores in 2014 and 2018 than those in lightly or not affected areas, but not in 2019 (Table 2).

**Table 2:** EQ-5D scores in years 2014, 2018 and 2019

	EQ-5D (2014)		EQ-5D (2018)		EQ-5D (2019)	
	Mean	(SD)	Mean	(SD)	Mean	(SD)
All	0.898	(0.178)	0.846	(0.191)	0.902	(0.163)
Full health, *n* (%)	794	(60.7)	528	(40.3)	652	(59.0)
Conflict level						
Not affected	0.924	(0.143)	0.868	(0.168)	0.906	(0.147)
Lightly affected	0.904	(0.170)	0.849	(0.189)	0.901	(0.166)
Heavily affected	**0.857**	(**0.215**)	**0.820**	(**0.214**)	0.899	(0.171)

Bold numbers indicate statistical significance at *p* = 0.05 level. People living in the heavily affected municipalities had statistically significant lower EQ-5D scores in 2014 and 2018 than those in not affected or lightly affected areas

### Validity analysis

Correlations between EQ-5D scores (2014, 2018 and 2019) and corresponding general health, mental health and disability scores were moderate to high (Table S2), supporting the convergent validity of EQ-5D for the population in our study. In the five known-groups, people with better (expected) underlying health status had statistically significant higher EQ-5D scores (Table S3), indicating that EQ-5D could distinguish the known-groups satisfactorily. These tests confirmed the validity of the EQ-5D instrument to assess HRQoL in this conflict-affected population.

### Panel data analysis

The panel data estimations indicate that living in a municipality lightly or heavily affected by conflict violence was consistently and negatively associated with HRQoL, compared to the HRQoL of people living in municipalities not affected (Table 3). The negative estimate for the association between living in a heavily affected area and a person’s HRQoL (coefficient: − 0.037) was roughly double that of living in an area lightly affected by conflict (coefficient: − 0.019). Compared to the mean EQ-5D score for people in non-affected municipalities in 2014, our estimates imply that EQ-5D scores were on average 4.0% lower for those in heavily affected municipalities and 2.1% lower for those in lightly affected municipalities. Moreover, people living in heavily affected areas tended to have poorer HRQoL than those in lightly affected areas, a difference that was at borderline statistical significance (*p* = 0.055).

**Table 3:** Results of the panel data analysis

	2014, 2018 and 2019	
	Coefficient	*p*-value
Year, 2014		
2018	**− 0.036**	** < 0.001**
2019	**0.023**	** < 0.001**
(2019 vs 2018)	**0.059**	** < 0.001**
Age group: ≤ 30		
31–55	**− 0.016**	**0.042**
> 55	**− 0.051**	** < 0.001**
Male	0.015	0.069
Ethnicity: white		
Black/Mestizo	0.017	0.095
Others	0.006	0.380
Marital: married/cohabiting		
Single	0.003	0.786
Separated/divorced/widowed	**− 0.020**	**0.013**
Education: primary/lower		
Secondary	**0.028**	** < 0.001**
Technical or higher	**0.032**	**0.001**
Occupation: employed		
Self-employed	**0.017**	**0.020**
Unemployed/others	− 0.008	0.326
Urban	− 0.005	0.504
Assets tertile: poor		
Middle	− 0.009	0.186
Rich	0.011	0.095
Health insurance: EPS (contributory)		
EPS (subsidised)	− 0.006	0.390
No insurance/others	− 0.005	0.608
Alcohol consumption (hazardous use)	0.014	0.096
Smoking (moderate to high dependency)	− 0.010	0.637
Hospitalisation	**− 0.088**	** < 0.001**
Sickness	**− 0.065**	** < 0.001**
Conflict level: no		
Lightly affected	**− 0.019**	**0.013**
Heavily affected	**− 0.037**	** < 0.001**
(heavily vs lightly)	− 0.018	0.055

Bold numbers indicate the coefficients that are statistically significant at *p* = 0.05 level

We also observed changing trends in HRQoL over time, with the EQ-5D score significantly reduced by 0.036 from 2014 to 2018 for our sample in general (3.9% lower compared to the mean EQ-5D score in 2014) and significantly increased by 0.059 from 2018 to 2019 (7.0% higher compared to the mean EQ-5D score in 2018) (Table 3).

Other factors associated with lower HRQoL included: older age, being separated or divorced or widowed, having only primary or lower education, and having worse underlying health status (proxied by having been hospitalised in the previous 12 months and having been sick in the previous 12 months) (Table 3).

Turning to differences in the associations between conflict exposure and HRQoL across specific subgroups, analyses including interactions terms between conflict level and relevant characteristics (sex, marital status and education) showed no statistically significant associations. The exception was that, for single people, living in a heavily affected area seems associated with an even worse HRQoL than for those people living in the same area who were married or cohabiting (at borderline statistical significance, *p* = 0.053; Table S4).

### Cross-sectional analysis

The negative association between living in areas affected by conflict and HRQoL in 2014 and 2018 was identified even more strongly in the corresponding year-by-year cross-sectional analyses, with coefficients larger in absolute magnitude than those in the panel data analysis, yet such association was not observed in 2019 (Table 4). In 2014, people in highly conflict-affected municipalities had statistically significant lower HRQoL than those in lightly affected municipalities, but this difference is not discernible in the 2018 or 2019 data.

**Table 4:** Results of the cross-sectional data analysis, for each data year

	2014		2018		2019	
	Coefficient	*p*-value	Coefficient	*p*-value	Coefficient	*p*-value
Age group: ≤ 30						
31–55	− 0.004	0.731	− 0.025	0.052	**− 0.027**	**0.009**
> 55	− 0.024	0.125	**− 0.068**	** < 0.001**	**− 0.058**	** < 0.001**
Male	0.015	0.160	**0.024**	**0.049**	− 0.008	0.539
Ethnicity: white						
Black/Mestizo	**0.033**	**0.016**	0.013	0.378	0.012	0.324
Others	0.008	0.436	0.007	0.527	0.008	0.425
Marital: married/cohabiting						
Single	0.023	0.123	0.016	0.341	− 0.041	0.083
Separate/divorced/widowed	**− 0.025**	**0.043**	− 0.001	0.937	**− 0.036**	**0.005**
Education: primary/lower						
Secondary	**0.028**	**0.010**	**0.044**	**0.001**	0.002	0.874
Technical or higher	0.022	0.104	**0.045**	**0.005**	0.007	0.638
Occupation: employed						
Self-employed	0.018	0.112	0.003	0.843	0.002	0.849
Unemployed/others	0.001	0.912	− 0.027	0.052	− 0.025	0.070
Urban	− 0.009	0.426	− 0.002	0.863	− 0.002	0.838
Assets tertile: poor						
Middle	0.001	0.962	− 0.002	0.895	− 0.007	0.594
Rich	0.005	0.646	**0.027**	**0.040**	**0.025**	**0.040**
Health insurance: EPS (contributory)						
EPS (subsidised)	− 0.006	0.614	0.00006	0.996	− 0.018	0.104
No insurance/others	0.012	0.460	− 0.002	0.926	− 0.032	0.108
Alcohol consumption (hazardous use)	0.021	0.101	0.0003	0.985	0.018	0.219
Smoking (moderate to high dependency)	− 0.048	0.110	− 0.002	0.959	0.034	0.212
Hospitalisation	**− 0.121**	** < 0.001**	**− 0.094**	** < 0.001**	**− 0.072**	** < 0.001**
Sickness	**− 0.085**	** < 0.001**	**− 0.085**	** < 0.001**	**− 0.056**	** < 0.001**
Conflict level: no						
Lightly affected	**− 0.027**	**0.009**	**− 0.026**	**0.027**	− 0.003	0.763
Heavily affected	**− 0.067**	** < 0.001**	**− 0.042**	**0.009**	0.002	0.893
(heavily vs lightly)	**− 0.040**	**0.0040**	− 0.016	0.259	0.005	0.684

Bold numbers indicate the coefficients that are statistically significant at *p* = 0.05 level

Other factors found to influence HRQoL were similar to those in the panel data analysis, and higher socioeconomic status (measured using assets) was also associated with better HRQoL in 2018 and 2019 (Table 4).

### Sensitivity analysis

Using EQ-5D value sets from other countries, the estimated associations between living in conflict-affected areas and people’s HRQoL were consistent with those in the main analyses, although slightly different in coefficient magnitudes using the value set for Chile (Table S5).

### QALY estimation

**Fig. 2:**
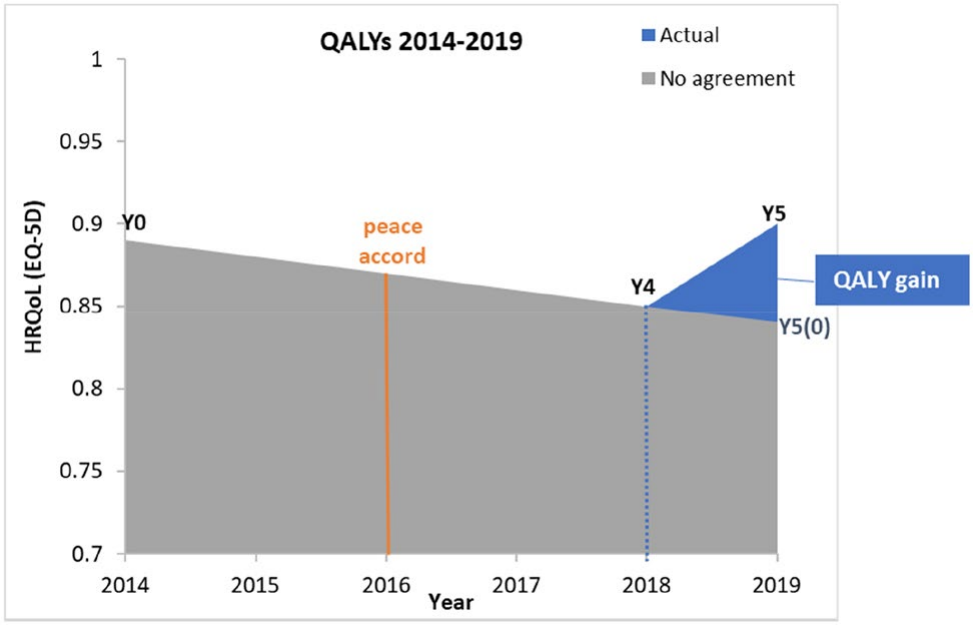
Quality-adjusted life years from 2014 to 2019

Under the (conservative) assumptions described previously, and illustrated in Fig. 2, the QALY gained since the peace accord up to year 2019 was 0.0343 per individual on average, equivalent to 0.41 month or 12.5 days in full health. Extrapolating this gain to all adults in the Meta province, the total QALY gain since the peace accord amounts to 20,752 QALYs.

## Discussion

In this study, we examined the validity of the widely used EQ-5D instrument to assess HRQoL in a conflict-affected population, explored the longitudinal correlations between conflict intensity levels and people’s HRQoL using both panel and cross-sectional data before and after the 2016 peace accord in Colombia, and identified the factors associated with the HRQoL of this conflict-affected population.

To our knowledge, this is the first study to assess the validity of EQ-5D in a conflict-affected population. Validity was assessed in two dimensions: convergent validity by testing its correlations with other health measures, and known-groups validity by exploring whether it could differentiate people differing in underlying health status. The results demonstrate that EQ-5D(-3L) is a feasible and valid instrument for the evaluation of HRQoL for this conflict-affected population in Colombia. As the SF-36 instrument used in other studies cannot directly provide utility scores for cost-effectiveness analysis, and the WHOQOL-BREF instrument was found to lack sensitivity in capturing changes in HRQoL among these people [15], the good validity results shown here support the wider use of EQ-5D for measuring HRQoL of conflict-affected populations, also permitting assessments of the impact of conflict on HRQoL and informing economic evaluations.

Another important innovation from our study is to uncover the existence (and magnitude) of longitudinal correlations between exposure to different degrees of conflict violence in the area of residence and people’s HRQoL, primarily through robust panel data estimation that allows controlling for several confounders over time. Our analyses found strong evidence—generalisable for an adult population of over half a million people, who lived under varying degrees of conflict violence—that the HRQoL of people living in conflict-affected areas was generally lower than that of people living in unaffected areas. This conclusion is in line with findings elsewhere [12, 13], although the differences and magnitudes estimated in our analyses represent more robust evidence than that in previous studies, which have suffered from methodological shortcomings including cross-sectional data restrictions and very limited ability to control for time-varying confounders, by using t-tests for mean differences or a single cross-sectional regression analysis.

We found that the difference in HRQoL between people living in heavily affected municipalities and those in unaffected municipalities (0.037) exceeded the minimum clinically important difference for the EQ-5D [30], which represents the minimal amount of impact that an individual would identify as important. This means that people’s HRQoL was impaired to a clinically important extent by conflict violence in the areas where they live. Importantly, taking together our panel data and separate cross-sectional estimations, we found supporting evidence for a dose–response relationship between conflict exposure and HRQoL: the more intense the conflict level in the area of residence, the worse a person’s HRQoL tends to be, after adjusting for many other characteristics. The cross-sectional analyses offer further insights about this relationship. For year 2014 (pre-peace accord), we found significant negative correlations between conflict levels and HRQoL (lightly affected: − 0.027; heavily affected: − 0.067); for 2018 (just after the peace accord), these correlations still existed but with a smaller magnitude (lightly: − 0.026, heavily: − 0.042); while for 2019, the correlations did not reach statistical significance, with very small point estimates. One plausible explanation for this pattern is that the Meta population started to benefit from improvements in quality of life brought about relatively soon after the general reduction in violence that followed the 2016 peace accord. The recovery of HRQoL in conflict-affected areas may have started around the peace accord in 2016, benefitting particularly those populations living in localities that were most affected by previous conflict violence, resulting in HRQoL differences that became indistinguishable across areas based on their pre-accord conflict levels, three years after the accord. Even under conservative assumptions that in practice consider HRQoL improvements since the peace accord occurring basically between 2018 and 2019, our back-of-the-envelope calculations suggest that such improvements may have translated into important QALY gains for this population already, potentially with additional “peace-time” gains accruing further down the line. Evaluating the causal effects of the peace accord itself on HRQoL is, however, beyond the scope of the present study, and further research is needed to scrutinise this topic in more depth.

As in previous studies [12–14, 31, 32], we examined and identified various factors influencing the HRQoL of conflict-affected individuals, including age, marital status, education, economic and health status. We found that older age was strongly associated with poorer HRQoL, with people aged above 55 years reporting significantly worse HRQoL than younger adults. In addition to the expected worsening in HRQoL as people age, this marked difference may also be attributable to the longer exposure to conflict among these older adults. People who were separated, divorced or widowed reported worse HRQoL than those married or cohabiting. We also found some suggestive evidence that, among people living in heavily conflict-affected areas, single people had even worse HRQoL than those married or cohabiting in the same areas. Overall, these findings could indicate that individuals with no cohabiting partners tend to suffer more from violence, potentially highlighting the importance of close family networks as a coping mechanism to protect the HRQoL of people exposed to intense conflict violence. Our findings that education, asset ownership and health status were positively associated with HRQoL may respond to issues such as limited supplies of food and water, and worse access to (quality) healthcare, among individuals with a lower socioeconomic status [31]. Taken together, the above results emphasise that certain population subgroups are likely to be more severely affected by long-lasting violence as it occurred in Colombia, offering policy-relevant insights about whom the most vulnerable groups may be and thus helping guide actions to protect and improve their HRQoL.

It should be noted that about 85% respondents (1106 out of 1309) in the first-round survey provided complete information in the follow-up survey. The full sample (*n* = 1309) and those followed-up (*n* = 1106) were comparable in observable characteristics (Table S1). We also performed a logistic regression analysis to identify any characteristics correlated with loss-to-follow-up, and found that participants being younger (30 years old or younger), single, or living in areas lightly affected by conflict violence were more likely to be lost to follow-up (Table S6). Nevertheless, these characteristics have been controlled for in our main regression analyses, so the loss-to-follow-up should not affect the reliability of associations drawn from our main estimates. Furthermore, we have re-run the panel analysis using data only for the 1106 participants observed in both data collection periods, and the results of this restricted analysis (shown in Table S7) were largely consistent with those from the main analysis. The only noteworthy difference in the restricted panel analysis is that being Black or Mestizo was found to be associated with higher HRQoL compared to people of white origin. In terms of the association between conflict levels and HRQoL, which is the main focus of this study, the existence and direction of the relevant associations identified in our main analyses remained unchanged, including for the presence of a dose–response relationship between conflict levels in the area of residence and HRQoL (in fact, if anything, the restricted panel data estimates suggest associations between conflict level and HRQoL that are even slightly *larger* in magnitude than in the main analysis).

An important limitation of this study is that the data for year 2014 (pre-peace accord) was collected retrospectively in 2018 (post-peace accord). Although many efforts were made in data collection to help people remember their socio-economic and health conditions in 2014 (see section ‘Methods’), it may be argued that recall bias cannot be completely ruled out from our analyses, since respondents may still not be able to fully recall their problems e.g. with respect to EQ-5D questions about anxiety/depression and pain/discomfort. Although we cannot completely discard this possibility with the data available, two factors help mitigate concerns about recall bias affecting our conclusions. First, as explained in Methods, we were able to implement recall tests using information collected in 2019, and the results of such tests suggested that any differences in recall ability across respondents—including for HRQoL questions—are uncorrelated with conflict levels where respondents lived (i.e., randomly distributed), hence allaying concerns about bias in the estimated associations between conflict levels and HRQoL. Second, even if there is unobserved recall bias affecting the 2014 responses, the most likely scenario is that this would be due to respondents overestimating their HRQoL in the pre-peace accord period, judged by the fact (perhaps counterintuitive) that mean EQ-5D scores were higher in the pre-peace accord period (0.898 in 2014) than those observed in the post-peace accord period (0.846 in 2018). This would imply that our baseline 2014 EQ-5D scores are higher than their “true” values, resulting in an underestimation of the associations uncovered in our work—i.e., our regressions would be providing conservative estimates of the gains in HRQoL post-accord for people living in conflict-affected areas. It must be noted, however, that at least part of the explanation for higher EQ-5D scores in 2014 than in 2018 may be the presence of genuinely better HRQoL on average for the study population in the former year. There is evidence that people exposed to long-term conflicts develop coping mechanisms to adapt to everyday routine in violent areas, in addition to benefitting in some cases from governance arrangements and networks sponsored by armed groups (including the provision by armed actors of some level of healthcare to the community, as it was the case with the FARC in Meta and other Colombian regions) [33, 34]. The implementation of the Colombian peace accord was not accompanied immediately by an increased presence of the State in conflict-affected areas, likely leading to disrupted coping mechanisms and networks amid a vacuum of authority and public service provision, as it has been observed in other conflict settings [35], which in turn might have harmed people’s HRQoL in conflict-affected areas shortly after the peace accord. Unfortunately, with our data we are unable to distinguish between the possibilities above.

Another limitation imposed by data availability is that the EQ-5D index scores were calculated using the value set for Argentina, in the absence of a value set for Colombia. When choosing the appropriate value set, we followed the guideline from EuroQol [36] to consider multiple factors such as geography (South America), language (Spanish), level of development of the country (low- and middle-income country). In South America, the EQ-5D-3L value set is available in Argentina, Brazil and Chile; from these options, Argentina is the country with strongest closeness to Colombia, with a Spanish-speaking population (unlike Brazil) and similar level of development (unlike high-income Chile) [37, 38]. In any case, as shown in the sensitivity analysis, whilst using the value set for Argentina, Brazil or Chile affects the specific point estimates, our general results about the presence, direction and magnitude of associations still hold regardless of the value set chosen, indicating that such choice is immaterial for the conclusions drawn from our study.

Lastly, our estimation of the QALY gains post-accord is only indicative. Due to the lack of information about how people’s HRQoL evolved year-on-year after the peace accord, we took the assumption that HRQoL started to improve only from 2018 onwards (Fig. 2). This is a conservative assumption because, in principal, some recovery in HRQoL could have started to take place earlier than 2018; if it was indeed the case, then the total QALY gain post-accord would be higher than we estimated here. Therefore, even though our study provides a novel attempt to quantify QALY gain in (post-)conflict or post-peace accord settings, our QALY estimates should be treated as preliminary only.

## Conclusion

The EQ-5D instrument appears to be valid to measure HRQoL for people affected by conflict violence, judged by our results for a conflict-affected population in Colombia. We observed a longitudinal dose–response relationship between conflict levels in the area of residence and people’s HRQoL, after taking into account several other factors. Our findings imply that especially vulnerable subgroups—e.g., people living in areas affected by severe chronic violence, and individuals with more limited family support networks—could be targeted by policy interventions to protect their HRQoL in conflict settings. Our findings also offer support to the likely positive impact of the Colombian peace accord—and, more generally, of reduced exposure to chronic violence—on peoples’ health. Future research seems warranted on causal mechanisms underlying these relationships and longer-lasting HRQoL impacts.

## Supplementary Information

Below is the link to the electronic supplementary material.

Supplementary file1 (DOCX 28 KB)

## Data Availability

The datasets generated and/or analysed during the current study are not publicly available due to constituting primary data collected by the research team, of which the research team has exclusive use for original research for three years after the end of the "War and Peace" research project in March 2021, as agreed with the project funder, UK Medical Research Council. We may make the data subset used in this paper available on reasonable request for the purposes of allowing replication of our results and research reproducibility.
