# Correction to: Health‑related quality of life of a conflict‑affected population in Colombia

**DOI:** 10.1007/s11136-021-02918-x

**Published:** 2021-07-07

**Authors:** Fan Yang, Sebastian Leon-Giraldo, Rodrigo Moreno-Serra

**Affiliations:** 1 Centre for Health Economics, University of York, York, UK; 2 Alberto Lleras Camargo School of Government and Interdisciplinary Centre of Development Studies, University of Los Andes, Bogotá, Colombia

## Correction to: Quality of Life Research https://doi.org/10.1007/s11136-021-02805-5

The article “Health‑related quality of life of a conflict‑affected population in Colombia”, written by Fan Yang, Sebastian Leon‑Giraldo and Rodrigo Moreno‑Serra, was originally published electronically on the publisher’s internet portal on 10 April 2021 without open access. With the author(s)’ decision to opt for Open Choice the copyright of the article changed to © The Author(s) 2021 and the article is forthwith distributed under a Creative Commons Attribution 4.0 International License, which permits use, sharing, adaptation, distribution and reproduction in any medium or format, as long as you give appropriate credit to the original author(s) and the source, provide a link to the Creative Commons licence, and indicate if changes were made. The images or other third party material in this article are included in the article’s Creative Commons licence, unless indicated otherwise in a credit line to the material. If material is not included in the article’s Creative Commons licence and your intended use is not permitted by statutory regulation or exceeds the permitted use, you will need to obtain permission directly from the copyright holder. To view a copy of this licence, visit http://creativecommons.org/licenses/by/4.0.

