# Supplementary material for: Health-related quality of life of a conflict-affected population in Colombia
Source: Qual Life Res. 2021 Apr 10;30(12):3559–69. doi: 10.1007/s11136-021-02805-5 (PMC8602178; doi:10.1007/s11136-021-02805-5)
Supplement: Supplementary file 1 — Supplementary file1 (DOCX 28 KB) [file 11136_2021_2805_MOESM1_ESM.docx]

# **Appendix**

**Table S1**. Characteristics of respondents in year 2018

|  | All  (n=1309) | | Excluding loss-to-follow-up  (n=1106) | |
| --- | --- | --- | --- | --- |
| Age at 2018, mean (SD) | 46.5 | (16.5) | 47.4 | (16.2) |
| Age group, n (%) |  |  |  |  |
| <=30 | 289 | (22.1) | 216 | (19.5) |
| 31-55 | 613 | (46.8) | 530 | (47.9) |
| >55 | 407 | (31.1) | 360 | (32.6) |
| Male, n (%) | 600 | (45.8) | 509 | (46.0) |
| Ethnicity, n (%) |  |  |  |  |
| White | 562 | (42.9) | 473 | (42.8) |
| Black/Mestizo | 217 | (16.6) | 192 | (17.4) |
| Others | 530 | (40.5) | 441 | (39.9) |
| Marital status, n (%) |  |  |  |  |
| Married/co-habiting | 817 | (62.4) | 697 | (63.0) |
| Single | 99 | (7.6) | 70 | (6.3) |
| Separated/divorced/widowed | 393 | (30.0) | 339 | (30.7) |
| Education, n (%) |  |  |  |  |
| Primary/lower | 614 | (46.9) | 550 | (49.7) |
| Secondary | 439 | (33.5) | 351 | (31.7) |
| Technical or higher | 256 | (19.6) | 205 | (18.5) |
| Occupation, n (%) |  |  |  |  |
| Employed | 470 | (35.9) | 406 | (36.7) |
| Self-employed | 314 | (24.0) | 260 | (23.5) |
| Unemployed/others | 525 | (40.1) | 440 | (39.8) |
| Urban, n (%) |  |  |  |  |
| Rural | 348 | (26.6) | 313 | (28.3) |
| Urban/town | 961 | (73.4) | 793 | (71.7) |
| Assets tertile, n (%) |  |  |  |  |
| Poor | 437 | (33.4) | 370 | (33.5) |
| Middle | 438 | (33.5) | 385 | (34.8) |
| Rich | 434 | (33.2) | 351 | (31.7) |
| Health insurance, n (%) |  |  |  |  |
| EPS (contributory) | 350 | (26.7) | 282 | (25.5) |
| EPS (subsidised) | 829 | (63.3) | 726 | (65.6) |
| No insurance/others | 130 | (9.9) | 98 | (8.9) |
| Hospitalisation in the last 12m, n (%) | 162 | (12.4) | 139 | (12.6) |
| Sickness in the last 12m, n (%) | 784 | (59.9) | 675 | (61.0) |
| Alcohol consumption, n (%) |  |  |  |  |
| Normal use | 1155 | (88.2) | 977 | (88.3) |
| Hazardous use | 154 | (11.8) | 129 | (11.7) |
| Smoking, n (%) |  |  |  |  |
| Low dependence | 1283 | (98.0) | 1083 | (97.9) |
| Moderate to high dependence | 26 | (2.0) | 23 | (2.1) |
| General health, n (%) |  |  |  |  |
| Poor | 123 | (9.4) | 108 | (9.8) |
| Fair | 577 | (44.1) | 504 | (45.6) |
| Good/very good/excellent | 609 | (46.5) | 494 | (44.7) |
| Mental health disorder, n (%) |  |  |  |  |
| <8, no | 885 | (67.6) | 749 | (67.7) |
| >=8, yes | 424 | (32.4) | 357 | (32.3) |
| Disability, n (%) |  |  |  |  |
| <17, no | 1046 | (79.9) | 873 | (78.9) |
| >=17, yes | 263 | (20.1) | 233 | (21.1) |
| Conflict level, n (%) |  |  |  |  |
| Not affected | 294 | (22.5) | 257 | (23.2) |
| Lightly affected | 709 | (54.2) | 570 | (51.5) |
| Heavily affected | 306 | (23.4) | 279 | (25.2) |

**Table S2**. Pearson’s correlation coefficients

|  | General health | Mental health score | Disability score |
| --- | --- | --- | --- |
| EQ-5D scores (2014) | **0.4101** | **-0.5711** | **-0.6491** |
| EQ-5D scores (2018) | **0.5454** | **-0.5765** | **-0.7037** |
| EQ-5D scores (2019) | **0.4564** | **-0.4766** | **-0.7044** |

Bold numbers indicate correlation coefficients statistically significant at p=0.05 level.

**Table S3**. Known-group comparisons

|  | Mean EQ-5D scores | | |
| --- | --- | --- | --- |
|  | 2014 | 2018 | 2019 |
| Age groups |  |  |  |
| <=30 years | **0.935** | **0.905** | **0.947** |
| 31-55 years | **0.902** | **0.859** | **0.914** |
| >55 years | **0.865** | **0.785** | **0.857** |
| Assets level |  |  |  |
| Low/middle | **0.888** | **0.825** | **0.887** |
| High | **0.916** | **0.890** | **0.930** |
| Hospitalisation in the previous 12 months | | | |
| No | **0.915** | **0.861** | **0.911** |
| Yes | **0.774** | **0.744** | **0.830** |
| Sickness in the previous 12 months | |  |  |
| No | **0.941** | **0.906** | **0.937** |
| Yes | **0.843** | **0.806** | **0.873** |
| Conflict level |  |  |  |
| No/lightly affected | **0.910** | **0.854** | - |
| Heavily affected | **0.857** | **0.819** | - |

Bold numbers indicate statistical significance at p=0.05 level.

**Table S4**. Results of panel data analyses including interactions terms

|  | #male | | #marital status | | #education | |
| --- | --- | --- | --- | --- | --- | --- |
|  | Coefficient | p-value | Coefficient | p-value | Coefficient | p-value |
| Year, 2014 as ref |  |  |  |  |  |  |
| **2018** | **-0.036** | **<0.001** | **-0.036** | **<0.001** | **-0.036** | **<0.001** |
| **2019** | **0.023** | **<0.001** | **0.002** | **<0.001** | **0.023** | **<0.001** |
| **(2018 vs 2019)** |  | **<0.001** |  | **<0.001** |  | **<0.001** |
| Age group: <=30 |  |  |  |  |  |  |
| **31-55** | **-0.015** | **0.044** | **-0.016** | **0.042** | **-0.016** | **0.040** |
| **>55** | **-0.051** | **<0.001** | **-0.051** | **<0.001** | **-0.051** | **<0.001** |
| Male | 0.009 | 0.503 | 0.014 | 0.080 | 0.015 | 0.068 |
| Ethnicity: white |  |  |  |  |  |  |
| Black/Mestizo | 0.017 | 0.095 | 0.017 | 0.090 | 0.016 | 0.102 |
| Others | 0.007 | 0.365 | 0.007 | 0.334 | 0.006 | 0.385 |
| Marital: married/cohabiting |  |  |  |  |  |  |
| Single | 0.003 | 0.794 | 0.030 | 0.116 | 0.002 | 0.829 |
| **Separate/divorced/widow** | **-0.020** | **0.013** | -0.025 | 0.082 | **-0.020** | **0.013** |
| Education: primary/lower |  |  |  |  |  |  |
| **Secondary** | **0.028** | **<0.001** | **0.027** | **<0.001** | 0.017 | 0.166 |
| **Technical or higher** | **0.032** | **0.001** | **0.032** | **0.001** | 0.019 | 0.245 |
| Occupation: employed |  |  |  |  |  |  |
| **Self-employed** | **0.017** | **0.022** | **0.017** | **0.016** | **0.016** | **0.022** |
| Unemployed/others | -0.009 | 0.304 | -0.008 | 0.346 | **-0.009** | 0.316 |
| Urban | -0.005 | 0.515 | -0.005 | 0.493 | -0.006 | 0.479 |
| Assets tertile: poor |  |  |  |  |  |  |
| Middle | -0.009 | 0.190 | -0.008 | 0.204 | -0.009 | 0.173 |
| Rich | 0.011 | 0.091 | 0.011 | 0.089 | 0.010 | 0.111 |
| Health insurance: EPS (contributory) |  |  |  |  |  |  |
| EPS (subsidised) | -0.006 | 0.379 | -0.006 | 0.412 | -0.006 | 0.382 |
| No insurance/others | -0.005 | 0.611 | -0.005 | 0.633 | -0.005 | 0.620 |
| Alcohol consumption (hazardous use) | 0.015 | 0.093 | 0.014 | 0.101 | 0.015 | 0.091 |
| Smoking (moderate-high dependency) | -0.010 | 0.632 | -0.012 | 0.591 | -0.010 | 0.632 |
| **Hospitalisation** | **-0.088** | **<0.001** | **-0.088** | **<0.001** | **-0.088** | **<0.001** |
| **Sickness** | **-0.065** | **<0.001** | **-0.065** | **<0.001** | **-0.065** | **<0.001** |
| Conflict level: no |  |  |  |  |  |  |
| **Lightly affected** | **-0.024** | **0.030** | **-0.018** | **0.035** | **-0.028** | **0.017** |
| **Heavily affected** | **-0.038** | **0.010** | **-0.037** | **0.003** | **-0.043** | **0.002** |
| (lightly vs heavily) |  | 0.302 |  | 0.096 |  | 0.214 |
| Lightly# male | 0.011 | 0.476 |  |  |  |  |
| Heavily# male | 0.001 | 0.954 |  |  |  |  |
| Lightly# single |  |  | -0.023 | 0.293 |  |  |
| Lightly# separate/divorce/widow |  |  | 0.002 | 0.913 |  |  |
| Heavily# single |  |  | -0.065 | 0.053 |  |  |
| Heavily# separate/divorce/widow |  |  | 0.016 | 0.442 |  |  |
| Lightly# secondary |  |  |  |  | 0.014 | 0.368 |
| Lightly# technical/higher |  |  |  |  | 0.021 | 0.287 |
| Heavily# secondary |  |  |  |  | 0.014 | 0.498 |
| Heavily# technical/higher |  |  |  |  | 0.004 | 0.871 |

Bold numbers indicate the coefficients that are statistically significant at p=0.05 level.

**Table S5.** Sensitivity analysis using alternative EQ-5D value sets

|  | 2014, 2018 and 2019 | | | | | | |
| --- | --- | --- | --- | --- | --- | --- | --- |
|  | Argentina (main analysis) | | Brazil | | | Chile | |
|  | Coefficient | p-value | | Coefficient | p-value | Coefficient | p-value |
| Conflict level: no |  |  | |  |  |  |  |
| **Lightly affected** | **-0.019** | **0.013** | | **-0.018** | **0.033** | **-0.026** | **0.010** |
| **Heavily affected** | **-0.037** | **<0.001** | | **-0.035** | **0.001** | **-0.046** | **0.001** |
| (heavily vs lightly) |  | 0.055 | |  | 0.087 |  | 0.101 |

Bold numbers indicate the coefficients that are statistically significant at p=0.05 level.

**Table S6**. Results of the logistic regression of loss-to-follow-up

|  | All (n=1309) | |
| --- | --- | --- |
|  | Coefficient | p-value |
| Age group: <=30 |  |  |
| **31-55** | **-0.619** | **0.002** |
| **>55** | **-0.719** | **0.005** |
| Male | 0.149 | 0.433 |
| Ethnicity: white |  |  |
| Black/Mestizo | -0.120 | 0.636 |
| Others | 0.275 | 0.112 |
| Marital: married/cohabiting |  |  |
| **Single** | **0.546** | **0.042** |
| Separated/divorced/widowed | 0.133 | 0.497 |
| Education: primary/lower |  |  |
| Secondary | 0.355 | 0.091 |
| Technical or higher | 0.950 | 0.715 |
| Occupation: employed |  |  |
| Self-employed | 0.237 | 0.293 |
| Unemployed/others | 0.220 | 0.319 |
| Urban | 0.239 | 0.273 |
| Assets tertile: poor |  |  |
| Middle | -0.377 | 0.071 |
| Rich | -0.092 | 0.671 |
| Health insurance: EPS (contributory) |  |  |
| EPS (subsidised) | -0.353 | 0.075 |
| No insurance/others | 0.325 | 0.207 |
| Alcohol consumption (hazardous use) | 0.036 | 0.888 |
| Smoking (moderate to high dependency) | -0.137 | 0.833 |
| Hospitalisation | -0.040 | 0.873 |
| Sickness | -0.064 | 0.698 |
| Conflict level: no |  |  |
| **Lightly affected** | **0.489** | **0.022** |
| Heavily affected | -0.246 | 0.403 |

Bold numbers indicate the coefficients that are statistically significant at p=0.05 level.

**Table S7**. Results of the panel analysis using data only for respondents observed both in 2018 and 2019

|  | All  (n=1309) | | Excluding lost to follow-up (n=1106) | |
| --- | --- | --- | --- | --- |
|  | Coefficient | p-value | Coefficient | p-value |
| Year, 2014 |  |  |  |  |
| **2018** | **-0.036** | **<0.001** | **-0.030** | **<0.001** |
| **2019** | **0.023** | **<0.001** | **0.026** | **<0.001** |
| **(2019 vs 2018)** | **0.059** | **<0.001** | **0.056** | **<0.001** |
| Age group: <=30 |  |  |  |  |
| **31-55** | **-0.016** | **0.042** | -0.014 | 0.095 |
| **>55** | **-0.051** | **<0.001** | **-0.044** | **<0.001** |
| Male | 0.015 | 0.069 | 0.007 | 0.456 |
| Ethnicity: white |  |  |  |  |
| Black/Mestizo | 0.017 | 0.095 | **0.022** | **0.037** |
| Others | 0.006 | 0.380 | 0.010 | 0.185 |
| Marital: married/cohabiting |  |  |  |  |
| Single | 0.003 | 0.786 | -0.005 | 0.661 |
| **Separated/divorced/widowed** | **-0.020** | **0.013** | **-0.023** | **0.007** |
| Education: primary/lower |  |  |  |  |
| **Secondary** | **0.028** | **<0.001** | **0.026** | **0.001** |
| **Technical or higher** | **0.032** | **0.001** | **0.032** | **0.001** |
| Occupation: employed |  |  |  |  |
| **Self-employed** | **0.017** | **0.020** | **0.020** | **0.009** |
| Unemployed/others | -0.008 | 0.326 | -0.015 | 0.108 |
| Urban | -0.005 | 0.504 | -0.007 | 0.375 |
| Assets tertile: poor |  |  |  |  |
| Middle | -0.009 | 0.186 | -0.008 | 0.233 |
| Rich | 0.011 | 0.095 | 0.005 | 0.472 |
| Health insurance: EPS (contributory) |  |  |  |  |
| EPS (subsidised) | -0.006 | 0.390 | -0.008 | 0.291 |
| No insurance/others | -0.005 | 0.608 | -0.007 | 0.541 |
| Alcohol consumption (hazardous use) | 0.014 | 0.096 | 0.016 | 0.088 |
| Smoking (moderate to high dependency) | -0.010 | 0.637 | -0.013 | 0.584 |
| **Hospitalisation** | **-0.088** | **<0.001** | **-0.085** | **<0.001** |
| **Sickness** | **-0.065** | **<0.001** | **-0.064** | **<0.001** |
| Conflict level: no |  |  |  |  |
| **Lightly affected** | **-0.019** | **0.013** | **-0.022** | **0.008** |
| **Heavily affected** | **-0.037** | **<0.001** | **-0.042** | **<0.001** |
| (heavily vs lightly) | -0.018 | 0.055 | **-0.020** | **0.045** |

Bold numbers indicate the coefficients that are statistically significant at p=0.05 level.
